# Ant Colony Optimization Analysis on Overall Stability of High Arch Dam Basis of Field Monitoring

**DOI:** 10.1155/2014/483243

**Published:** 2014-06-17

**Authors:** Peng Lin, Xiaoli Liu, Hong-Xin Chen, Jinxie Kim

**Affiliations:** ^1^State Key Laboratory of Hydroscience and Engineering, Tsinghua University, Beijing 100084, China; ^2^Department of Civil and Environmental Engineering, The Hong Kong University of Science and Technology, Clear Water Bay, Hong Kong

## Abstract

A dam ant colony optimization (D-ACO) analysis of the overall stability of high arch dams on complicated foundations is presented in this paper. A modified ant colony optimization (ACO) model is proposed for obtaining dam concrete and rock mechanical parameters. A typical dam parameter feedback problem is proposed for nonlinear back-analysis numerical model based on field monitoring deformation and ACO. The basic principle of the proposed model is the establishment of the objective function of optimizing real concrete and rock mechanical parameter. The feedback analysis is then implemented with a modified ant colony algorithm. The algorithm performance is satisfactory, and the accuracy is verified. The *m* groups of feedback parameters, used to run a nonlinear FEM code, and the displacement and stress distribution are discussed. A feedback analysis of the deformation of the Lijiaxia arch dam and based on the modified ant colony optimization method is also conducted. By considering various material parameters obtained using different analysis methods, comparative analyses were conducted on dam displacements, stress distribution characteristics, and overall dam stability. The comparison results show that the proposal model can effectively solve for feedback multiple parameters of dam concrete and rock material and basically satisfy assessment requirements for geotechnical structural engineering discipline.

## 1. Introduction

Scale models [1, 2], intelligent cracking control [3–5], numerical simulation [6–8], and prototype monitoring structures [9–11] have been used to investigate the behavior of concrete arch dams and foundation for the last 80 years. The purpose of these investigations is to understand the behavior of a high arch dam and their foundations by studying the external and internal actions during operation period. Failures of large dams, such as at Malpasset, Vaiont, Teton, and Kolnbrein [12–15], can be highly catastrophic, posing a great danger to people and properties. Hence, much attention should be paid to long-term safe operation of dams based on prototype surveillance and inverse analysis.

Parameters such as elastic modulus, unit weight, Poisson's ratio, friction coefficient, and cohesion are parameters in structural analysis intrinsic to the determination of stress distributions and displacements, especially when the design of the structure is based on elasticity considerations. In a dam-foundation system, these parameters, for mass concrete, are hard to determine directly from tests due to the necessity for large specimens and large testing machines. The parameters for rock are also hard to determine because of the complicated nature of most geological situations. Currently, the inversed parameter is focused on Young's modulus of concrete and rock material, which may limit the variety of materials in the inversion Young's modulus. If we can find an algorithm with capability of inversion Young's modulus of more material, and even a variety of mechanical parameters of various materials, this will be a powerful tool for determining the mechanical parameters of dam-foundation systems. Through inverse analysis, the exact parameter values can be determined, and a precise evaluation of dam cracking mechanism, the overall stability of dams, and underground excavations can be made [16–20].

In recent years, inverse analysis has been mainly based on the two approaches of neural networks [21, 22] and optimization algorithms [23–27]. Three types of optimization algorithm have been used in feedback analysis. The first is the gradient-based direct search algorithm, such as the Levenberg-Marquardt method, conjugate gradient method, and thrust region method. The second is the relatively simple direct search method such as the simplex search method. The last type is the intelligent global search algorithm, such as genetic algorithms [24, 25], monkey algorithms [26, 27], differential evolution, particle swarm optimization, and ant colony optimization (ACO) [28, 29]. The first and the second types of algorithms both have the advantage of estimating solutions in relatively short computational times, but the results are affected by the initial values chosen and premature convergence is likely to occur. As an alternative to direct search algorithms, intelligent global search algorithms are being widely used for reverse analysis, but they are time-consuming. Ant colony optimization is one of the most widely used optimization algorithms, since it can avoid problems of premature convergence and is not influenced by the initial values [30, 31]. Ant colony optimization (ACO) is a metaheuristic algorithm for combinatorial optimization problems. The ACO algorithm was first introduced in the early 1990s [28, 32, 33] and was successfully applied to some academic problems and to real-word applications [34–36]. The ACO algorithm has been used for inverse analysis by some researchers investigating the material parameters of dams and embankment [29]. Several extensions and improvements of the original ant system (AS) algorithms have been introduced over the years. These successful ACO variants include elitist AS (EAS), rank-based AS (RAS), MAX-MIN ant system (MMAS), ant colony system (ACS), and hypercube framework (HCF). However, successful application of the ACO algorithm in the case of real large arch dams has been rather limited. The objective of this study was to develop a modified ACO algorithm for determining the mechanical material parameters of a large arch dam based on the monitored deformation data.

The paper is organized as follows. Firstly, the modified ACO algorithm for inverse analysis is proposed. Secondly, based on actual operational conditions and the monitored Lijiaxia arch dam deformation data collected over decades [37, 38], inverse analysis of the dam was carried out by employing the FEM code [39]. The optimized mechanical parameters of concrete and rock of the dam and abutments were found to be obtainable through this process. Finally, 3D numerical analysis of the entire dam was carried out using these optimized parameters to evaluate the effects of reinforcement in the dam and to investigate the crack initiation mechanism, how cracks propagated in the downstream face of the dam, and the overall safety of the dam foundation.

## 2. Nonlinear Feedback Numerical Model

### 2.1. Numerical Inverse Analysis of a Large Arch Dam

Structural health monitoring of large concrete arch dams is based on the acquisition of displacement measurements. These displacements are interpreted to identify significant deviations from what could be considered as the normal response based on statistical or deterministic models of dam behavior.

The finite element analysis method is adopted for solving the dam-foundation system. The analytical model in finite element formulation is

(1) $$\begin{array}{c} \left[K\right]\left\{u\right\}=\left\{F\right\}, \end{array}$$

where K is the structural stiffness matrix, uis the displacement vector, and F is the load vector. Given K and F, ucan be obtained. Generally, F is known beforehand; K depends on parameters such as Young's modulus, cohesion, and Poisson's ratio.

Actual deformations of arch dams can be obtained through monitoring. Given a group of concrete-rock mechanical parameters, displacements can be computed by (1). The parameters should be optimized to match the real scenario by minimizing the objective function, which is expressed as the sum of the squares of the errors between the computed displacements and the field monitored displacements. The mathematical model for inverse analysis of arch dams can be expressed as

(2) Min⁡T(p)=∑i=1k[ai(P)−ai∗]2subjected  to [K]{u}={F}  pj−≤pj≤pj+, (j=1,2,…,n),

where *P* is the mechanical parameters vector; *n* is the total number of observation points; *a*_i_* is the monitored displacement at the *i*th observed point; and *a*_*i*_(*P*) is the corresponding numerical analysis displacement at the *i*th monitoring point.

### 2.2. Parameter Inverse Analysis Model

This study is similar to the application of ACO in the case of travelling salesman problem (TSP) [30, 40]. Inverse analysis in the search for mechanical dam foundation parameters can be defined as follows.

There are *n* parameters and *m* ants. Since ACO is good at solving discretized optimization problems [36], the parameters are discretized first. Each parameter has a range and can be divided into some segments. Then the center of each segment is represented by a point. For example, the elastic modulus, *E*, typically ranges from 1 to 50 GPa and 100 points can be selected within this range. Similarly, 100 points for each of *μ*, *γ*, *f*, and *c*, which are Poisson's ratio, unit weight, friction coefficient, and cohesion coefficient, can be selected. More points mean higher accuracy but with more computation time. The goal is to find a group of parameters that contains every parameter and minimizes the value of *T*(*P*). If there is more than one kind of material involved, the number of parameters increases.

*P* is the vector of the mechanical parameters to be analyzed, the dimension of which is *n*. *S* is the search space of *P*, which is defined as

(3) $$\begin{array}{c} S=\left\{P_{i}\;|\;p_{i-}\leq p_{i}\leq p_{i+},\;\;\left(i=1,2,\ldots ,n\right)\right\}, \end{array}$$

where *P*_*i*_ is the *i*th mechanical parameter of *P*. The difference between *P*_*i*−_ and *P*_*i*+_ defines the range of the *i*th mechanical parameter.

An artificial ant is an agent which moves between parameter points. It chooses the next point by using a probabilistic function determined by both the pheromone value, *τ*, and the heuristic value, *η*, which is the standard deviation.

*η* is defined as

(4) ηij(t)=∑w,w⊃(i,j)[Tw(p)−E(Xij)]2gij +a×ηij(t−(n−1)),

where *a* is a constant which ranges from 0 to 1 and *n*is the number of parameters. Every ant has a tour. Tour *w* corresponds to *T*_*w*_(*P*). If tour *w* includes edge (*i*, *j*), it can be described as *w*⊃(*i*, *j*). *g*_*ij*_ represents the number of ants that choose edge (*i*, *j*). *E*(*X*_*ij*_) is the average value of all the *T*_*w*_(*P*) relating to ants choosing edge (*i*, *j*). It is given as

(5) $$\begin{array}{c} \eta_{ij}E\left(X_{ij}^{t}\right)=\frac{\sum_{w,w⊃(i,j)}T_{w}\left(p\right)}{g_{ij}\left(t\right)}, \end{array}$$

where *g*_*ij*_(*t*) reflects the sensitivity of the edge (*i*, *j*) and must be updated. Faster convergence is prevented if more ants have a higher probability of choosing those more sensitive edges.

Without *a* × *η*_*ij*_(*t* − (*n* − 1)), if the number of ants choosing edge (*i*, *j*), *η*_*ij*_(*t*) is zero, the search space is smaller and smaller. Thus, the move probability from site-*i* to site-*j* for ant-*k* at time *t* can be described by

(6) $$\begin{array}{c} p_{{}_{ij}}^{k}=\{\begin{array}{cc} \frac{\underset{\;\;\;\;\;\;\;\;\;\;\;\;\;\;\;\;\;\;\;\;\;\;\;\;\;\;\;\;\;\;\;\;\;\;\;\;\;\;\;\;\;\;\;\;\;\;\;\;\;\;\;\;\;\;\;\;\;\;\;\;}{{\left[\tau_{ij}\left(t\right)\right]}^{\alpha }{\left[\eta_{ij}\left(t-\left(n-1\right)\right)\right]}^{\beta }}}{\sum_{k\in \text{allowe}{\text{d}}_{k}}\left[{\left[\tau_{ik}\left(t\right)\right]}^{\alpha }{\left[\eta_{ik}\left(t-\left(n-1\right)\right)\right]}^{\beta }\right]}+h & \\ \;\;\;\;\;\;\;\;\;\;\;\;\;\text{if}\;j\in \text{allowe}{\text{d}}_{k}, & \\ 0\;\;\;\;\;\;\;\;\;\;\;\;\;\;\text{otherwise}, & \end{array} \end{array}$$

where *h* is a constant, which ensures that edges with zero transition probability also have a poor chance of being selected by the ants.

Additionally, *τ* can be updated as

(7) $$\begin{array}{c} \tau_{ij}\left(t+n-1\right)=\rho \tau_{ij}\left(t\right)+\Delta \tau_{ij}\left(t\right),\;\forall \left(i,j\right)\in L, \end{array}$$

where *ρ* is the pheromone evaporation rate which is a constant parameter and ranges between zero and one. Consider

(8) $$\begin{array}{c} \Delta \tau_{ij}\left(t\right)=\overset{m}{\underset{k=1}{\sum }}\Delta \tau_{ij}^{k}\left(t\right),\;\forall \left(i,j\right)\in L\\ \Delta \tau_{ij}^{k}=\{\begin{array}{cc} \frac{Q}{L_{k}} & \text{if}\;\text{ant-}k\;\text{edge}\left(i,j\right)\;\text{is}\;\text{in}\;\text{its}\;\text{tour}\\ 0 & \text{otherwise}, \end{array} \end{array}$$

where *Q* is a constant parameter and *L*_*k*_ is the tour length of ant-*k*. The combination of *α*, *β*, *ρ*, and *Q* should be determined to be the specific case:

(9) $$\begin{array}{c} L_{k}=T_{k}\left(P\right)+g, \end{array}$$

where *T*_*k*_(*P*) is the *T*(*P*) value corresponding to ant-*k* and *g* is a constant to avoid the overflow of Δ*τ*_*ij*_^*k*^ when *T*_*k*_(*P*) is too small.

### 2.3. Nonlinear Constitutive Model

The characteristics of high arch dams and dam foundations lead to 3D complex mesh model, highly nonhomogeneous material distributions, and very high loadings. All of these have detrimental impacts on the convergence of elastic-plastic analyses. The convergence of FEM is a principal characteristic of a stable geotechnical structure, for example, the specific and widely used strength reduction method. In this study, the back-analysis adopted a nonlinear constitutive model based on the Drucker-Prager (D-P) criterion [41, 42]. A brief introduction is as follows.

In this study, robustness of iteration and an integration policy based on the D-P criterion increased the stability of calculation. This method improved computational convergence and ensured that computation converged to the correct solution. The yielding condition for the ideal elastic-plastic model adopted the D-P criterion:

(10) $$\begin{array}{c} f=\alpha I_{1}+\sqrt{J_{2}}-k\leq 0, \end{array}$$

where

(11) $$\begin{array}{c} I_{1}=\sigma_{1}+\sigma_{2}+\sigma_{3}\\ J_{2}=\frac{1}{6}\left[{\left(\sigma_{1}-\sigma_{2}\right)}^{2}+{\left(\sigma_{2}-\sigma_{3}\right)}^{2}+{\left(\sigma_{3}-\sigma_{1}\right)}^{2}\right], \end{array}$$

where *σ*_1_, *σ*_2_, and *σ*_3_ are major principal stresses, respectively. *I*_1_ is the first invariant stress, *J*_2_ is the second invariant stress, *α* and *k* can be obtained by the fitting Mohr-Coulomb (MC) yielding criteria. On the *π* plane, if the D-P criterion is the circle circumscribing the Coulomb hexagon, then

(12) $$\begin{array}{c} \alpha_{1}=\frac{2\sin\varphi }{\sqrt{3}\left(3-\sin\varphi \right)},\;\;k_{1}=\frac{6c\cdot \cos\varphi }{\sqrt{3}\left(3-\sin\varphi \right)}, \end{array}$$

where *φ* and *c* are friction angle and cohesion coefficient of the material, respectively. If the D-P criterion is the inscribed circle to the Coulomb hexagon, then

(13) $$\begin{array}{c} \alpha_{2}=\frac{2\sin\varphi }{\sqrt{3}\left(3+\sin\varphi \right)},\;\;k_{2}=\frac{6c\cdot \cos\varphi }{\sqrt{3}\left(3+\sin\varphi \right)}. \end{array}$$

In this study

(14) $$\begin{array}{c} \alpha =\frac{1}{2}\left(\alpha_{1}+\alpha_{2}\right),\;\;k=\frac{1}{2}\left(k_{1}+k_{2}\right). \end{array}$$

Concrete and rock masses are materials low in tensile strength. Conditions of tension are

(15) $$\begin{array}{c} \sigma_{1}\leq \sigma_{t},\;\;\sigma_{2}\leq \sigma_{t},\;\;\sigma_{3}\leq \sigma_{t} \end{array}$$

(16) $$\begin{array}{c} \sigma_{t}=\frac{c}{\tan\varphi }, \end{array}$$

where *σ*_*t*_ is the uniaxial tension strength of the materia. If the tension strength is not given, it is evaluated according to (16) to determine its tensile strength. The program flow, first of all, estimates whether the tension condition is satisfied. If not, stresses are adjusted until the condition is satisfied. The stresses are again estimated after the adjustment.

The literature [23, 39] shows that the analytic solution for transferred stress based on the D-P criterion is equivalent to a linear prediction-radical adjustment algorithm as far the stress adjustment process is concerned. It is equivalent to the so-called closest projection algorithm from the aspect of constitutive relationships integration policy. The closest projection algorithm is of first-order accuracy and is stable unconditionally. As a special case of the generalized midpoint method, it can achieve high accuracy over large strain increments.

### 2.4. Modified ACO Inverse Analysis for Large Arch Dam

To verify the accuracy of the method, a group of parameters are chosen to obtain computed displacements employing the FEM code [39]. These computed displacements can be viewed as the measured ones. The inverse displacements are then compared with prototype monitored values.

**Figure 1:**
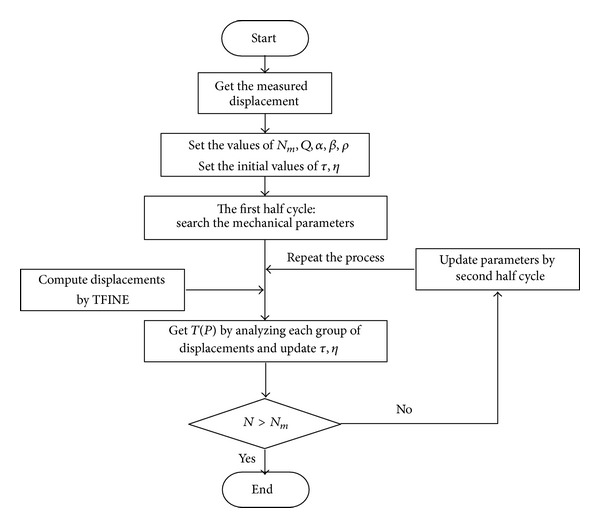
Flow chart of D-ACO inverse analysis for deformation of large arch dam.

In this modified ACO model, let artificial ants search such that each ant will have a tour which contain a group of parameters. An ant cycle has two half-motions. Particularly, in first half-motion, the artificial ants start at random points between *P*_1−_ and *P*_1+_. Once the artificial ants arrive at *P*_*n*_, they then choose points to move to in tracing a path towards *P*_1_, which is the second half-motion, completing the ant cycle. The probability of ant choosing the next point is determined by *τ*_*ij*_ and *η*_*ij*_. The initial values of pheromone between the points are the same. The same is true of *η*. Thus, in the first half-motion, parameter values are unlike those applying to the prototype of the ACO algorithm. Obviously, once the first half-motion is accomplished, then *m* groups of parameters will be obtained which are used to run the FEM code to obtain numerical displacements. Then, *τ* and *η* will be updated. Importantly, the second half-motion is not random. Only the first half-motion of the first cycle is random. The procedure above is repeated until a satisfactory result is obtained. The procedure of ACO process is presented in Figure 1. The key concept and parameters of the modified ACO model correspond with the arch dam and are descripted in Table 1.

## 3. Case Study

### 3.1. Brief of Lijiaxia Power Station

**Figure 2:**
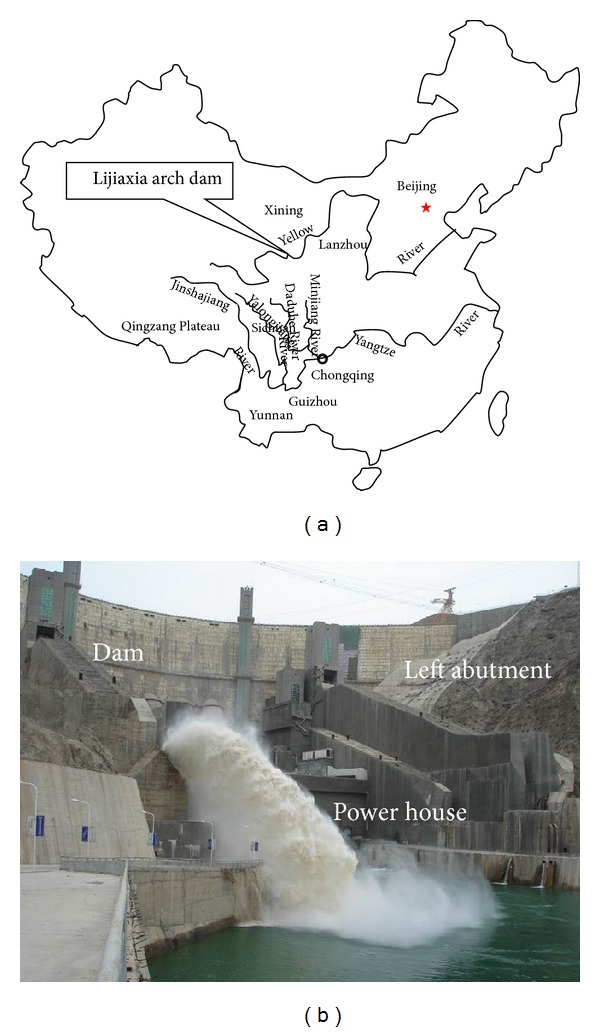
Location map and snapshot of the Lijiaxia arch dam: (a) schematic map of the location; (b) a snapshot of Lijiaxia arch dam.

Lijiaxia hydropower station is located on the Yellow River, border between the villages of Jianzha and Hualong in Qinghai province, about 100 km southeast of Xining (Figure 2(a)). The dam is a concrete double curvature arch dam with maximum height, 155 m. The elevation level (EL) of the dam crest is at 2185 m and the maximum crest width is 45 m (Figure 2(b)). The total installed capacity is of 2,000 MW. The Lijiaxia arch dam has multiple functions including the provision of hydropower, flood control, and irrigation. A gravity block was installed at the left abutment, and a discharge structure, a station diversion system and a power house structure of units in two rows were constructed at the dam downstream, as shown in Figure 2(b). The dam is EL 2185 m, as stated above, and the normal reservoir water surface is EL 2180.0 m. The storage volume is 16.5 hundred million m^3^. Project construction began in April 1988 and the reservoir began impounding on December 26, 1996. The dam has been operating safely after about twenty years.

**Figure 3:**
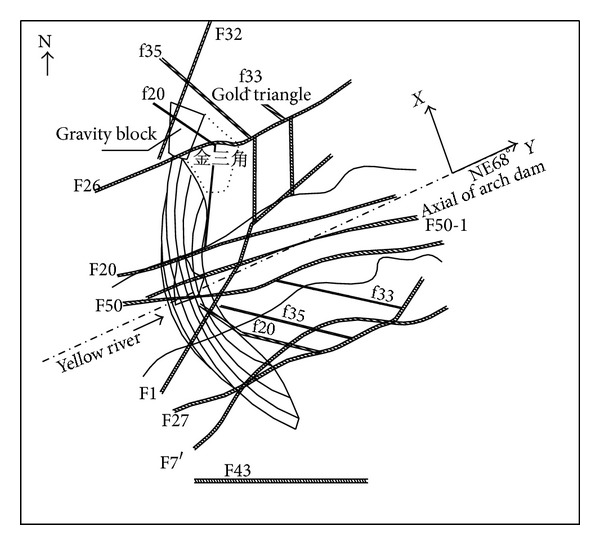
Faults distribution in dam zone of Lijiaxia arch dam [38].

The dam lies across the middle of the Lijiaxia Valley, about 5 km long, a deep, narrow, and V-shaped gorge. The valley is also symmetrical with a slope angle for two side abutments of 45~50°. The dam foundation is very complicated, and the bedrock consists of simian black mica schist and chlorite schist in continuous bands interlocked with granite rock. The joints are comparatively well developed. The metamorphic rock at the dam-heel is influenced by multistage tectonic activity, and faults and joints are well developed; see Figure 3.

### 3.2. Numerical Model and Analysis Cases

**Figure 4:**
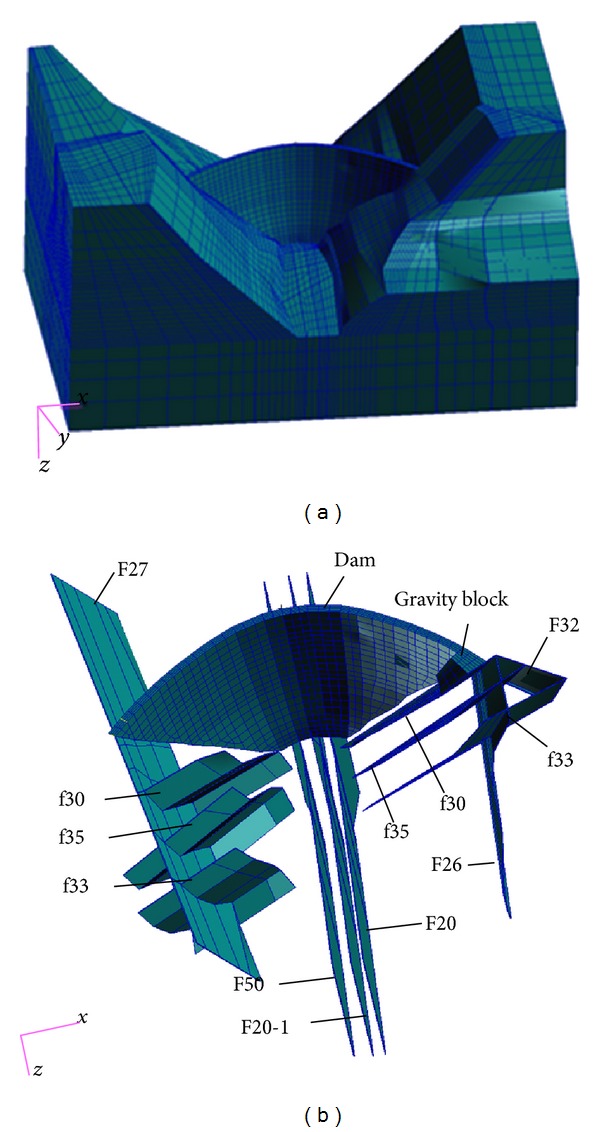
3D FEM mesh model of Lijiaxia arch dam: (a) 3D overall mesh model; (b) main faults distribution.

On the basis of the actual Lijiaxia project sites (Figure 2), a 3D finite element model was constructed as in Figure 4(a). The 3D finite element model includes the whole arch dam (Figure 4(b)) and the foundation, which includes the main faults shown in Figure 3. The size of the 3D numerical model represents 480 × 700 × 305 m (length × width × height), which is much bigger than the dam size itself, since a vast area of the abutments is included in the model. All of the rock masses together with the related geological structures are represented in the 3D numerical model. The rock masses are of types A2, A4, A1, B2, B4, C3, and D around the dam, and under the dam are of types A2, A4, A1, B2, and B4. The geological structures in these ranges include faults f33, f20, f35, F32, F26, F27, F20, F50, and F201, as shown in Figures 3 and 4(b). The main elastic modulus parameters of the rock masses, of the geological structures (i.e., weak zones), and of the arch dam material are listed in Table 2 based on the monitoring feedback analysis. The total number of hexahedral elements was 25376 and 29529 of these were used to model the dam and foundation in Figure 4(a).

During analysis, the node displacements of the overall model are applied as the boundary conditions. The upstream/downstream surfaces of foundation are employed displacement constraints along the river direction (*Y*) and constraint of two abutment side surfaces, using a transverse river direction to the (*X*). The bottom surface of foundation is taken as the vertical displacement constraints (*Z* direction).

The numerical analysis especially takes reinforcement parameters into account. Analysis and evaluation mainly concerns dam displacements, stresses, safety failure locations in the dam model and foundation, stability evaluation of the abutments, and the riverbed interface. In this study, the dam self-weight, water and silt loadings, and temperature loadings were taken into account in the analyses. The upstream water level is EL 2178 m and the downstream level is EL 2050 m. The main analysis cases are listed below.

 Analysis case 1: dam self-weight + normal water load.
 Analysis case 2: dam self-weight + normal water load + silt load + temperature dropped loading (the temperature loading determined by the average March temperature).
 Analysis case 3: dam self-weight + normal water load + silt load + temperature increased load (the temperature loading determined by the average September temperature).

In order to prove the ant colony optimization analysis is effective in relation to the monitored feedback as far as concrete and rock mechanical parameters are concerned, a comparison analysis was carried out employing various material parameters obtained from design values, generalized least squares, and neural networks methods.

### 3.3. Feedback Analysis on Material Parameter

#### 3.3.1. D-ACO Initial Value Determination

According to the proposed inverse analysis model in Section 2.2, each group of parameters corresponds to a group of displacements, and these displacements are defined as computed data. As the value of each parameter is continuous, some discrete points are chosen within the range. In the inverse analysis program, the number of discrete points for each parameter is 110. For the inverse analysis, it is better to set 1.5 as the ratio of site to ant number, and the ant number is chosen as 10. In addition, it was found that the control values are very important in the D-ACO algorithm, and it is very essential to determine them by test computing. In this case, the values of control parameters were set as *m* = 50, *α* = 1, *β* = 2, and *ρ* = 0.7.

#### 3.3.2. D-ACO Pheromone Update

This is the updating stage of the pheromone value, *τ*, and the heuristic value, *η*, following the first ant cycle. The object of pheromone updating is to study the influence of material parameters on the D-ACO algorithm. The heuristic algorithm parameters are *τ* = 1, *σ* = 20, and *Q* = 300. According to the proposed feedback model in Section 2, three times the discrete space is constructed for solving material parameter feedback of Lijiaxia arch dam.

#### 3.3.3. Established of the Objective Function

Based on discussion of Section 2.2, comparing site monitoring and numerical displacements, the objective function is established as shown in (2) in Section 2.1 above. Then, the minimized objective function is then determined.  Table 3 shows the field monitoring and numerical displacements used for solving the objective function *T*(*P*).

#### 3.3.4. Parameter Feedback Results

In this study, the elastic modulus, *E*, for thirteen material types was inversed. The feedback analysis results for the various material parameters are shown in Table 2.

The feedback results show (1) an elastic modulus of the dam concrete which is about 36% greater; (2) an elastic modulus of abutment and riverbed rock 4~85% which were not included feedback in the comparisons. Based on feedback results shown in Table 2, the D-ACO feedback parameter results are better than for the other two methods.

### 3.4. Feedback Analysis Results Discussion

Based on the material parameters obtained from the various methods (original design value, generalized least squares), comparative analyses were conducted on dam displacements, stress characteristics, and overall stability of dam-foundation system.

#### 3.4.1. Comparison on Dam Displacement

Table 4 shows the main numerical displacement results under case 2.  Table 5 shows the displacement differences in the various numerical results using various feedback material parameters. Based on the Tables 4 and 5 results, the largest dam displacement along the direction of the river decreased under analysis case 2. Comparing with that simulated using original design values, the difference *δY* is 9.5 mm (Table 5). The abutment displacements in the direction along the river also show decreasing values of the left and right hand abutments of 1.68 mm and 2.27 mm, respectively. These values are basically equivalent.

Under the same analysis case, at the upper EL 2050 m level, in the direction perpendicular to the river, the difference in displacements between design material parameters and optimized material parameters (D-ACO) is greater on the right side than on the left abutment. Below EL 2050 m, the difference in displacement of right side is greater than that of a shift toward the left side. The results are consistent with field monitoring results. The displacements monitoring of each dam monolith showed a shift to the right bank before 1998, while in 1998, and especially after 2000, except for the foundation displacement which continued to shift toward the right bank, the displacement of the upper part of the arch crown shifted towards the left bank. The shifted displacements were 1.53 mm at EL 2150 m and 2.45 mm at EL 2185 m.

**Figure 5:**
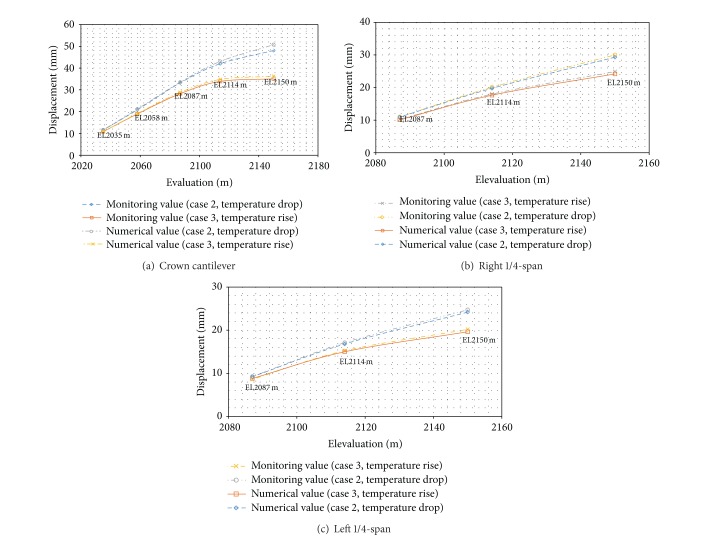
Numerical and field survey results of dam displacement in the direction along river.

Figure 5 shows numerical and monitoring displacements of the crown cantilever, at the left and right hand span of the dam. The comparison results are consistent.

Contrasting curves of temperature rise and fall at different elevation levels for each dam monolith are shown in Figure 5. The numerical results show the following.

1. Field monitoring results and feedback analysis results fit particularly well in the arch crown beam and right hand arch (approximately 1/4 arch, number 6 dam monolith). Monitored results and feedback analysis for the temperature drop condition (case 2) are basically the same.
2. Errors exist between field monitoring results and feedback evaluation at EL 2150 m in the left hand arch (approximately 1/4 arch results, number 16 dam monolith).
3. Generally after ten years dam prototype observations, monitoring and reinforcement numerical analysis of the dam downstream are basically the same.

#### 3.4.2. Comparison of Dam Stresses

**Figure 6:**
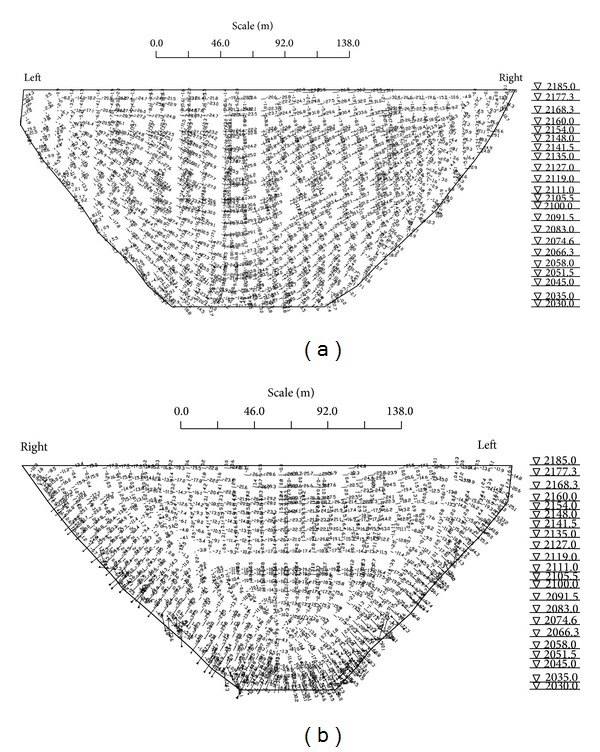
Principal stress vector contour of upstream/downstream surface under analysis case 2: (a) upstream surface; (b) downstream surface.

For analysis case 2, stress distributions (Figure 6) on both upstream and downstream dam faces satisfy the usual stress regularity of a double curvature arch dam. The largest compressive stresses are between 3.3~3.6 MPa on the downstream face and 1.2~2.6 MPa tensile on the upstream face. Numerical results show that the tensile stress on the upstream surface is greater than that on the downstream face. The stress distribution characteristics with various calculated material parameters are shown in Table 6.

The comparison analysis is as follows.

1. Under analysis case 2, the dam downstream surface is predominantly in a compressive stress state adopting the optimal parameters evaluation. The maximum compressive stress when using D-ACO parameters is −7.22 MPa, greater than the cases of design parameters and generalized least squares.
2. Analyzing the two cases of temperature drop and temperature rise, there is a tensile zone in the same direction as the beam at the dam downstream surface. The stress level is below 1 MPa in both cases.
3. The dam is in a stable stress state. Comparing the results obtained with different numerical material parameters, the characteristic of dam stress is in agreement with that obtained by D-ACO.
4. The comparison results show that the improved ant colony algorithm (D-ACO) can effectively determine the material mechanical parameters and reflects well the actual dam deformation and dam stress distribution. The algorithm accuracy satisfies project safety evaluation requirements.

#### 3.4.3. Comparison on Overall Safety Factors

By employing the material parameter obtained from D-ACO and generalized least squares, the overall dam safety factors are shown in Table 7. Based on comparison results, the following conclusions are illustrated.

1. Under analysis case 2, dam foundation safety factors are basically symmetric. After reinforcement, point safety factor at the elevation EL 2150 m at the left bank is a little lower than that at the right. No overall yielding appears.
2. The riverbed always has the lowest safety factors, and cracks first occur at the dam-foundation interface. Once yielding zones and cracks occur, unbalanced thrust forces will transfer to those zones with comparatively higher safety factors at both abutments, where high bearing capacity levels develop. As the height-width ratio of Lijiaxia arch dam is high, and there is a large pedestal of thickness 30 m, the safety factor in the valley upstream is at least 2.0 in any case under normal load, and there is no cracking.
3. During dam overloading process, unbalanced forces in the arch dam will transfer to the two banks, under the water load of 3 times normal loads, the depth of the crack upstream is about 1/4 of the dam thickness, and the point safety factor is about 1.0~2.0. The carrying capacity of the riverbed will reduce and transfers to areas with higher safety factors at both banks. Under a water load of 3 times normal loads, the dam can still work and without any yielding zone at the downstream interface. Under a water load of 5 times, there is yielding at the downstream interface.
4. Based on Table 7, the overall safety factor if using D-ACO parameters is slightly lower than with the case of generalized least squares. Abutment safety factors are reduced by approximately 5% to 15%.

Above all, modified ACO showed effectively distributed computing capabilities, strong robustness, and easiness to combine with other algorithms, or FEM numerical code, and can well avoid premature convergence phenomenon. The proposal model can effectively solve for feedback multiple parameters of dam concrete and rock material. Through inverse analysis, the exact parameter values can be determined, and a precise evaluation of dam cracking and deformation mechanism and the overall stability of dam-foundation can be made.

## 4. Conclusions

Resulting from this study, a modified dam ant colony optimization (D-ACO) model is proposed for obtaining concrete and rock mechanical parameters of a large arch dam. Based on field monitored deformations and the ant colony optimization technique, a typical dam parameters feedback problem was solved using a nonlinear back-analysis numerical model. The basic D-ACO principle is introduction of distributed computing, D-ACO initial value determination, and D-ACO pheromone update, which is used to establish the objective function. The numerical analysis is implemented through proposed dam ant colony algorithm. The mechanical parameters are determined using this algorithm, and construct solutions combined with nonlinear constitutive relations.

By employing the proposed back analysis model, calculated deformations and a stability evaluation of the Lijiaxia arch dam were compared with the monitoring results taken over 10-year supervision period of the dam prototype. The results demonstrated that the proposed model can effectively solve for feedback multiple parameters of dam concrete and rock material. Through inverse analysis, the exact parameter values can be determined, and a precise evaluation of dam cracking and deformation mechanism and the overall stability of dam-foundation can be made.

## Figures and Tables

**Table 1:** The key concept and parameters of the ACO model corresponding with the arch dam.

Concept	Define corresponding with the arch dam
Parameter discrete	Each group of concrete or rock mechanical parameters corresponds to a group of displacements and these displacements are defined as computed data. As the value of each parameter is continuous, some discrete points are chosen within the range.

Ant	An ant is an agent which moves between parameter discrete points. Each ant can choose a group of parameters during each search.

*T*(*P*)	Objective function is expressed as the sum of the squares of the errors between the computed displacements and the field monitored displacements. *P* is the vector of the mechanical parameters to be analyzed.

Pheromone update	The object of pheromone updating is to study the influence of material parameters on the modified ACO algorithm.

Inversion parameter	To find a group of parameters that contains every parameter and minimizes the value of *T*(*P*).

Edge (*i*, *j*)	Relationship of the discrete points between two adjacent parameters, for example, each of 50 discrete points of two adjacent parameters, there are 2,500 edges between parameters.

One cycle	Including two searches: “moveahead” and “moveback,” and output is two times parameters

One tour *w*	A group of parameters, the tour is optimized depending on *T*(*P*), average value of all the *Tw*(*P*) relating to ants choosing edge (*i*, *j*)

**Table 2:** The elastic modulus inversion results using various feedback methods (unit: GPa).

Number	Material	Design value	DPIP-ACO	Generalized least squares	Neural networks
1	Dam concrete	20	27.2	30	28
2	A2	20	23.3	28	26 (A)
3	A4	12	11.2	17	
4	B2	8	10	11.6	22 (B)
5	B4	12	16.4	18	
6	C3	5	7	7	13 (C)
7	A1	15	16	22.5	26 (A)
8	F20	0.62	—	—	—
9	F20-1	0.31	—	—	—
10	F26	0.52	—	—	—
11	F27	0.57	—	—	—
12	F32	0.243	—	—	—
13	F50	0.21	—	—	—
14	D	2.75	3.4	3.85	—
15	f20	0.94	—	—	—
16	f33	1.05	—	—	—
17	f35	0.8	—	—	—
18	Gravity pier	20	29.6	30	—
19	Foundation reinforcement zone	10	27.2	15	—

**Table 3:** Field monitored displacement values and numerical results for solving the objective function *T*(*P*).

Monitoring point	EL (m)	Dam monolith	Displacement of case 3 (mm)		Displacement of case 2 (mm)	
			Monitoring	Numerical simulation	Monitoring	Numerical simulation
2	2150	Number 6	19.52	17.75	24.33	25.1
3	2114	Number 6	15.64	15.05	17.71	18.6
4	2087	Number 6	7.29	10.75	8.45	12.2
5	2150	Number 11	25.53	23.7	33.42	38
6	2114	Number 11	26.84	24	29.74	32
7	2087	Number 11	22.22	20	22.87	25
8	2059	Number 11	13.65	14	15.30	16
9	2035	Number 11	8.18	7.8	8.74	8.8
10	2150	Number 16	12.91	19.1	14.05	25.8
11	2114	Number 16	12.20	14.2	15.32	17.1
12	2087	Number 16	8.08	8.3	9.05	9.3

**Table 4:** Dam displacements in the direction along/perpendicular the river (analysis case  2, unit: mm).

Analysis case	EL (m)	Design value						Generalized least squares						D-ACO					
		Right arch side		Arch crown		Left arch side		Right arch side		Arch crown		Left arch side		Right arch side		Arch crown		Left arch side	
		*Y*	*X*	*Y*	*X*	*Y*	*X*	*Y*	*X*	*Y*	*X*	*Y*	*X*	*Y*	*X*	*Y*	*X*	*Y*	*X*
Case 2	2185	1.5	0.7	48.8	1.3	3.4	1.4	0.9	−0.4	35.0	0.9	2.2	0.9	0.9	−0.7	39.6	0.7	2.6	1.7
	2148	4.5	−2.6	48.6	1.2	5.3	2.4	3.0	−1.7	34.1	0.8	3.6	1.7	2.6	−1.8	37.9	0.6	3.9	2.5
	2119	7.3	−3.3	42.8	1.0	8.0	3.0	5.0	−2.2	30.1	0.7	5.6	2.2	4.5	−2.3	33.2	0.5	5.9	2.8
	2100	8.7	−3.5	37.3	0.7	9.3	3.2	6.1	−2.4	26.2	0.5	6.6	2.4	5.6	−2.6	28.7	0.3	7.0	3.0
	2075	10.4	−3.3	27.7	0.5	8.5	2.6	7.3	−2.3	19.4	0.4	6.1	2.0	7.4	−3.0	21.0	0.2	6.8	2.9
	2050	9.4	−2.0	17.9	0.3	8.4	2.5	6.7	−1.4	12.6	0.2	6.0	1.9	6.6	−2.0	13.4	0.1	6.5	2.6
	2035	7.7	−0.9	11.4	0.2	7.3	1.8	5.6	−0.6	8.2	0.2	5.3	1.4	5.5	−1.1	8.6	0.1	5.6	1.8
	2030	6.9	−0.4	9.4	0.2	6.8	1.5	5.0	−0.3	6.8	0.2	4.9	1.1	5.2	−0.9	7.2	0.1	5.3	1.4
	Max. value	**10.4**	**−3.5**	**49.1**	**1.4**	**9.3**	**3.2**	**7.3**	**−2.4**	**35.0**	**0.9**	**6.6**	**2.4**	**7.4**	**−3.0**	**39.6**	**0.7**	**7.0**	**3.0**

*X* represents displacements in the direction perpendicular the river; positive value means deformation to left abutment; *Y* represents displacements in the direction along the river; and positive value means deformation to downstream direction.

**Table 5:** The difference of downstream displacement in the direction perpendicular, along river obtained from various feedback analysis methods (analysis case  2, unit: mm).

Analysis case	EL (m)	Between generalized least squares and design						Between D-ACO and design					
		Right arch side		Arch crown		Left arch side		Right arch side		Arch crown		Left arch side	
		*δY*	*δX*	*δY*	*δX*	*δY*	*δX*	*δY*	*δX*	*δY*	*δX*	*δY*	*δX*
Case 2	2185	0.6	1.1	13.8	0.4	1.2	0.5	0.6	1.4	9.2	0.6	0.8	−0.3
	2148	1.5	−0.9	14.5	0.4	1.7	0.7	1.9	−0.8	10.7	0.6	1.4	−0.1
	2119	2.3	−1.1	12.7	0.3	2.4	0.8	2.8	−1	9.6	0.5	2.1	0.2
	2100	2.6	−1.1	11.1	0.2	2.7	0.8	3.1	−0.9	8.6	0.4	2.3	0.2
	2075	3.1	−1	8.3	0.1	2.4	0.6	3	−0.3	6.7	0.3	1.7	−0.3
	2050	2.7	−0.6	5.3	0.1	2.4	0.6	2.8	0	4.5	0.2	1.9	−0.1
	2035	2.1	−0.3	3.2	0	2	0.4	2.2	0.2	2.8	0.1	1.7	0
	2030	1.9	−0.1	2.6	0	1.9	0.4	1.7	0.5	2.2	0.1	1.5	0.1
	Max. value	**3.1**	**−1.1**	**14.1**	**0.5**	**2.7**	**0.8**	**3**	**−0.5**	**9.5**	**0.7**	**2.3**	**0.2**

**Table 6:** Characteristic value of dam stresses under analysis case  2.

Position	Stress type	Design (MPa)	Generalized least squares (MPa)	D-ACO (MPa)
Upstream surface	Maximum tensile stress	1.24 (left side)	1.35 (left dam abutment)	2.56 (left side)
	Maximum compressive stress	−3.6 (EL 2100 m)	−3.67 (EL 2100 m)	−3.33 (EL 2093 m)

Downstream surface	Maximum tensile stress	0.61 (EL 2150 m)	0.69 (EL2141 m)	0.7 (EL 2151 m)
	Maximum compressive stress	−7.52 (EL 2060 m)	−7.73 (EL 2060 m)	−8.12 (EL 2060 m)

Interface	Maximum tensile stress	0.61 (dam heel)	0.38 (dam heal)	—
	Maximum compressive stress	−1.87 (EL 2030 m)	−1.88 (EL 2030 m)	—

**Table 7:** Point safety factor of abutments.

EL (m)	Generalized least squares		D-ACO	
	Left abutment	Right abutment	Left abutment	Right abutment
2185	3.0 (gravity pier), 1.5~2.0 (out of gravity pier)	2	2.0~3.0 (gravity pier); 1.1~1.5 (out of gravity pier)	2.0

2148	1.5~3, 1.2~1.5 (fault)	2~3	1.5~3.0; 1.0~1.5 (fault)	1.5~2.0

2100	2~3	2, 1.2~1.5 (fault)	1.5~3.0	1.5~2.0; 1.2~1.5 (fault)

2050	2~3, 1.2~1.5 (fault)	2	2.0~3.0; 1.1~1.5 (fault)	1.5~2.0

2030	2 ~ 3	2~3	1.5~3.0	1.5~5.0
